# Selections and Abstracts

**Published:** 1913-10

**Authors:** 


### NEWS AND NOTES—SELECTIONS AND ABSTRACTS

### FIRST ANNUAL MEETING OF THE GEORGIA SURGEONS' CLUB, WEDNESDAY AND THURSDAY, NOVEMBER 5TH AND 6TH, 1913, AT ATLANTA, GA.

### CLINICS

WEDNESDAY, NOVEMBER 5TH.

8:30-10:30—Dr. G. H. Noble, Grady Hospital—Gynecological.

8:30-10:30—Dr. Jas. L. Campbell, Atlanta Medical College—Surgical.

11:00-12:00—Dr. E. G. Ballenger, Atlanta Medical College—Administration of Salvarsan.

10:30-1:00—Dr. F. K. Boland, Grady Hospital—Surgical.

10:30-1:00—Dr. J. N. Ellis, Grady Hospital—Surgical.

10:00-12:00—Dr. Willis Jones, Wesley Memorial Hospital—Surgical.

2:00-4:00—Dr. W. F. Westmoreland, Atlanta Medical College—Surgical.

4:00-6:00—Dr. Michael Hoke and Dr. F. G. Hodgson, Grady Hospital—Orthopedic.

WEDNESDAY EVENING, NOVEMBER 5TH.

6:30—Subscription Dinner, $2.00 per plate—Assembly Room, Hotel Ansley.

8:00—Business Meeting—Adoption of Constitution and By-Laws, Election of Officers, Etc.

9:00—Symposium—"The Necessity of an Early Exploratory Incision in Obscure Abdominal Conditions."—Hotel Ansley Assembly Room.

Symptoms relative to the

(a)—Stomach, pylorus and duodenum—Dr. W. F. Westmoreland, Atlanta, Ga., Dr. G. R. White, Savannah, Ga.

(b) Gall-bladder and liver—Dr. G. H. Noble, Atlanta, Ga., and Dr. W. W. Battey, Jr., Augusta, Ga.

(c) Caecum and appendix—Dr. F. W. McRae, Atlanta, Ga., and Dr. H. S. Munroe, Columbus, Ga.

Also symptoms relative to the

(d) Kidney, ureter and bladder—Dr. J. N. Ellis, Atlanta, Ga., and Dr. C. C. Harrold, Macon, Ga.

### CLINICS

THURSDAY, NOVEMBER 6TH.

8:30-10:30—Dr. F. W. McRae, Piedmont Sanatorium—Surgical. (By invitation.)

8:30-10:30—Dr. S. T. Barnett, Grady Hospital—Gynecological.

8:30-10:30—Drs. W. F. Shallenberger and Montague Boyd, office Candler Building—Cystoscopic Demonstration. (By invitation.)

10:30-12:30—Dr. E. G. Jones, Grady Hospital—Surgical.

10:30-12:30—Dr. F. K. Boland, Grady Hospital—Surgical.

11:00-1:00—Dr. A. L. Fowler, Atlanta Medical College—Genito-Urinary.

2:00-4:00—Dr. J. G. Earnest, Grady Hospital—Gynecological.

2:00-4:00—Dr. L. Sage Hardin, St. Joseph's Infirmary—Surgical. (By invitation.)

2:00-4:00—Dr. L. C. Fischer, Davis-Fischer Sanatorium—Surgical. (By invitation.)

3:00-5:00—Dr. W. B. Emery, Wesley Memorial Hospital—Genito-Urinary.

4:00-6:00—Dr. Michael Hoke and Dr. F. G. Hodgson, Grady Hospital—Orthopedic.

### OFFICERS

E. C. Davis, M. D., Atlanta, Ga., President; Thos. J. McArthur, M. D., Cordele, Ga. Vice-President; R. M. Harbin, M. D., Rome, Ga., Secretary-Treasurer.

EXECUTIVE COMMITTEE—W. S. Goldsmith, M. D., Atlanta, Ga., Chairman; G. R. White, M. D., Savannah, Ga., W. W. Battey, Jr., Rome, Ga.

Local Committee on Arrangements—Dr. E. C. Davis, Dr. W. S. Goldsmith, Dr. W. S. Elkin, Dr. W. F. Westmoreland Dr. F. W McRae, Dr. W. A. Crowe, Dr. W. C. Jarnigan, Dr. G. H. Noble, Dr. J. G. Earnest, Dr. J. N. Ellis.

Headquarters—Hotel Ansley.

---

Editor Journal-Record of Medicine:

Owing to the unforeseen circumstances, the date for clinic days in Atlanta for the Georgia Surgeons Club has been changed from October 15-16 to November 5-6, 1913, (Wednesday and Thursday). The following committee of arrangements instructs the Secretary to make this announcement: Drs. W. S. Goldsmith, Chairman, W. S. Elkin, W. P. Nicholson, F. W. McRae, W. A. Crowe, J. N. Ellis, Geo. H. Noble, W. F. Westmoreland, J. G. Earnest, W. C. Jarnigan and E. C. Davis.

It is being arranged to occupy the entire days of November 5-6 with surgical clinics in the various hospitals of Atlanta and the character of the heads of these clinics bespeaks a profitable trip to all of those in attendance who are interested in the study of surgery. In the evening of Nov. 5th there will be a business meeting of the Club, after which a symposium on the "Diagnostic Methods to be Employed in the Treatment of Abdominal Emergencies", the leaders in the discussion to be announced later. At this meeting plans for visiting clinics in other cities will be discussed.

The Club now has an enrollment of 59 members and this circular is being mailed to an incomplete list of those who are especially interested in surgery throughout the state, among the membership of the Medical Association of Georgia.

The only qualification for membership at present is membership in the State Association, and rules governing such qualifications will be enacted at the forthcoming meeting.

A complete program of the clinics, along with other announcements, will be made later.

If you are interested in raising the standard of surgery and hospitals in Georgia, and establishing some systematic means for the clinical study of surgery, send your name and address to the Secretary who will gladly accept any suggestions to be put before the meeting for discussion, and state if you can attend the Atlanta meeting.

Yours truly,

R. M. HARBIN, Sec.-Treas.

---

### MEETING OF THE FULTON COUNTY MEDICAL SOCIETY.

SEPTEMBER 4, 1913.

R. R. DALY, M. D.

Dr. Hansell Crenshaw read a paper on "The Diagnosis of Insanity" in which he took up the most modern view-points of classification and diagnosis.

Dr. J. N. Brawner said that it is very important to differentiate between legal and medical insanity. The standards are different and many are abitrary and confusing. There is, in the first place, no positive definition for either sanity or insanity, so one must determine each case by a careful study of it with plenty of time for observation.

The sending of relatives to an institution for the care of the insane is a big problem, but nowadays it had better be done early than to wait till great damage has been done. Institutions now do not suffer under the classic reproach of holding sane patients unlawfully. If a patient is admitted and soon shows sanity or that there was no real insanity at the time of examination, no harm has been done and the patient is as promptly dismissed as if the occurrence were in a general hospital for some other trouble.

Recurrent attacks of real insanity are very trying because there are intervals of soundness of mind that make it appear strange to the layman that the patient should have to be held in a hospital at all. But still more troublesome than these are the cases of paranoia with their systematized delusions and their tendency to homicide.

Dr. W. A. Gardner said that medico-legal cases are the hardest to diagnose because of the varying standards in different states.

Malingering, both positive and negative, is an interesting and trying condition to recognize. One dealing with insane, and especially the medico-legal type, has always to keep this in mind. Various forms of delirium stimulate insanity and require some little time for observation.

Dr. J. C. King said that insanity is a disease of the brain and should be so considered and treated always. The forms of idiocy and imbecility that are either congenital or due to lack of development are outside this discussion.

In many cases of insanity, memory, consciousness and even reason are not dethroned, and yet the sufferer has not the power to choose or do the right.

Dr. A. W. Stirling read a paper on "Early Treatment of Some Diseases" which appears in the Journal.

Dr. R. R. Daly said that children could not "outgrow" anything that was wrong and that they were often cursed by this particular form of ignorance or laziness on the part of their guardians. If for example, a child could not see during its early years and were allowed to go beyond puberty with defective vision, it had irrevocably lost the impressions that correct and complete vision should have given during the formative years. Its tendency then was to introspection because by lack of vision it was cut off from the sports and normal associations of childhood. The same thing is true of leaving tonsils and adenoids to interfere with normal hearing, and of nasal obstructions that interfere with proper breathing and the cleansing of the blood dependent upon good breathing. Sucn a child was always morbid in some degree and the type of morbidity became almost invariably the sexual. The reason for this is plain when one considers the dominance of this appetite over every other function unless it is restrained. The straint comes in childhood through giving the growing child all the opportunity for play and happy exercise of its faculties with other children that it can have. Shut the child off from any of these by deferring the remedy of diseases of any of the special senses through which it should learn the world, and you have a perverted child.

Dr. F. P. Calhoun said that he had noticed in his service in Grady Hospital that there had been a marked lessening of mastoid cases since the school examinations of children had commenced. He attributed this to the knowledge the parents gained as to the children's condition and to remedies they were urged to apply in cleaning the throats. He spoke of a case of pyorrhoea aveolaris and terrible tonsils that had been allowed to persist in a child till iritis and rheumatism had supervened.

Dr. Stirling in closing confirmed Dr. Calhoun as to the lessening of the ear cases in the Grady Hospital.

Dr. G. M. Niles read a paper on "The Stomach Tube" in which he demonstrated its use and value in diagnosis and treatment of gastric diseases.

---

### REGULAR MEETING, FULTON COUNTY MEDICAL SOCIETY.

---

SEPTEMBER 18, 1913.

R. R. DALY, M. D.

Dr. R. R. Daly read a paper on "Non-Surgical Treatment of the Accessory Sinuses" which appears elsewhere in the Journal. Dr. R. M. Nelson said in discussing it that he was satisfied that the sinuses could be reached in the manner described and that good would result in many cases, but that as to his personal experience in an attack of hay fever, he had secured no relief.

Dr. Daly said that hay fever in the acute stage was entirely out of the realm of his paper and beyond the reach of any remedy he knew of, but that when the sinuses were thoroughly cleansed for weeks before the attacks came on, relief had frequently been given.

Dr. E. C. Thrash read a paper on "The Treatment of Tuberculosis with Schafer Vaccine" in which he stated that he had been successful in a number of properly selected cases and was encouraged to make further investigations as to the somewhat questioned remedy.

Dr. L. C. Rouglin said that more cures were recommended in tuberculosis than in any other disease and that anything that came at all well recommended would have great use for a time. The very large number of tubercular cases and the variable progress they made would lead naturally to this state of affairs. He said that the "aqueous solution of the metabolic processes of germs" was not a vaccine. It was claimed to be an antigen but really it is a toxin. Its use has not been sustained by scientific men and it is dangerous unless given under the strictest precaution and observation. The product is put forth commercially and not under scientific, laboratory clinical methods. He commended Dr. Thrash for his care in investigating the substance, but he did not foresee a successful future for it.

Dr. S. W. Merritt said he liked to hear of new things coming up for the treatment of tuberculosis in which he is especially interested, but he has not as yet found anything equal to fresh air, good food and proper hygienic attention. He hopes for success from vaccines and suggests that the autogenous ones are more likely to be of avail.

Dr. S. R. Roberts said that he was especially interested in Dr. Thrash's paper and its subject matter. One must approach it with a humble and scientific spirit and should know all about it there is to learn before discussing it, but whatever the method, we must all bow to the results obtained. Thus he has to commend Thrash's work because of the improvement in the patients as described, but personally, he places Shafer's Vaccines in the same category as Diamond Dyes. There is the rheumatism one for that disease, the tubercular one for its diseases and so on just as you go to the store and get pink or blue to dye the cloth the color you want. As for the phrase "Aqueous solution of the metabolic processes of bacteria" he would like to know what it could mean. It seemed to him to be irreparably indefinite. Scientific men are all against phylacogens. The finding of a germ and using it autogenously is a rational procedure but the phylacogens are shot gun doses.

Dr. A. H. Bunce said that he had seen the cases mentioned in the paper and good results had certainly followed the treatment. We can not pretend to have cure for the tuberculosis in Shafer's vaccine, but where there is a mixed infection as is the case in nearly all tubercular patients, the vaccine does stimulate the antigens and assist the natural reparative processes to the recovery of the patient. He had tried to make autogenous vaccines in every case and learned that it was a mistake. There is always mixed infection and it is impossible to say in just what proportion the mixture of the various germs occurs in the system. Thus it is quite as accurate to use the phylacogen as it could possibly be to make autogenous vaccines.

Dr. H. I. Battey said that he was in Dr. Billings' clinic in Chicago recently and saw many cases of autogenous vaccines but the patients were not doing well.

Dr. Thrash said in closing that he commenced the use of these vaccines in 1912. The earlier cases all improved. Later on he learned that he must select the cases that would accept the treatment. As to the vaccine, itself, it is not a commercial product and not on the market. It is in the experimental stage and he has been using it in that way and studying it scientifically. It will be marketed if the results coming from careful men all over the country seem to warrant it. It is not a shot gun remedy, it is a metabolic product because it is the product of the germs as they are grown in an aqueous medium. That is plain and simple and can not be anything else.

As to the name "vaccine". In the common acceptance of speech today, it is a vaccine, it stimulates the antibodies and whatever will o this is a vaccine. The word is a misnomer in that is comes from "vacca" a cow, and this was first used in connection with the small pox vaccine.

Wherever the tubercular process by ulceration or otherwise, comes in contact with the air, there is mixed infection and this is always in variable and indeterminable quantities, thus autogenous vaccine is uncertain.

### REGULAR MEETING FULTON COUNTY MEDICAL SOCIETY.

OCTOBER 2, 1913.

R. R. DALY, M. D.

Dr. L. M. Gaines read a paper on "Diagnostic Significance of Convulsions" that appears elsewhere in the Journal.

Dr. E. Bates Block said that this was a great subject and that in discussing it, it was hard to determine where to begin or where to leave off. Between sixty and seventy diseases have convulsions as symptoms and in not all of these are the convulsions distinctive. Among children having convulsions, one in eight cases are epileptic. Dentition is prolific of convulsions and in teething children this cause is looked for more than epilepsy and may mask it. However, the frequency, and the character of the spasm will make the differentiation if carefully watched. Adherent prepuces will often cause convulsions and there are cases where children have suffered for years until relief has been afforded by proper operation on the fore-skin.

After the age of thirty, convulsions are rarely of epileptic origin. They are induced by some organic disease, such as tumor or cerebral syphilis.

The so-called functional or idiopathic diseases that have been given as causing convulsions are gradually and surely yielding to investigation and disclosing actual conditions that are amenable to treatment. The part that syphilis plays is becoming better known and many convulsions are now cured this time that syphilis was the etiologic factor in General Paresis.

Dr. R. R. Daly said that he was interested in the essayist's remarks about cerebral syphilis. It used to be thought that all cases of General Paralysis were of syphilitic origin, but that he had seen several cases in which no syphilitic history could be found. This was before the discovery of the Spirocheta pallida, however, he asked what evidence there was at that syphilis was the etioligic factor in General Paresis.

Dr. Gaines said in closing that it was impossible to do more than touch upon the outskirts of this subject in one paper. National ideas concerning convulsions were interesting. For instance, the English deny to dentition the importance we ascribe to it. They say that the convulsions coming at that time are due to Ricketts and point to the poor nutrition as the cause of the disturbance as well as of the poor teething.

As to the cause of Paresis, he said that small trephinings have been done in the living patients and the living spirochetes have been demonstrated in the cortical substance. Besides this, in a large percentage of the post mortem cases, the dead spirochetes have been found and it is reasonable to assume that they are present in all cases. They are not numerous at best and may be overlooked in the apparently negative cases.

Dr. W. F. Shallenberger read a paper on "Some Diseases of the Female Urethra", which appears elsewhere in this Journal. Dr. J. G. Earnest in discussion of the paper said that we have reflex trouble as a frequent cause of urethral difficulty and that fissure of the anus often operated in this way.

As to Skene's glands, he had seen many more than the essayist had mentioned as being present. He has a case at present in which there are at least seven distinct openings. They are inflamed but responding to the nitrate of silver treatment.

Dr. Shallenberger said in closing that reflex cases do arise and that prolapsed kidneys and hydronephrosis were among them. When he said there were two or three Skene's glands in the urethra, he was stating the standard or normal number. There were anomalies such as Dr. Earnest mentioned when many more appeared.

Dr. J. H. Neall read a paper on "Hydrotherapy at Home" which appears elsewhere in this Journal.

Dr. S. H. Lindmore said that he approved the methods as set forth in the essay. The cold girdle had been of great assistance to him in treating dysentery. It soothed the patient and allowed him to get along without opiates. He had other experiences in which the pack did not work so well.

In children suffering with pneumonia and dry, persistent cough, the cold, wet pack around the chest had been very beneficial. Care should be taken to have the dry cloth or flannel extend well over the wet application and this must be kept dry. Great relief has been given by this means of treatment to spasmodic croup.

Dr. L. L. Andrews said he heartily subscribed to Dr. Neall's paper. Circulation can be controlled in this way and once we have gained that advantage, the other matters are comparatively easy. Convulsions in children can be controlled by the continued warm bath as in no other way. Typhoid fever is best treated by hydrotherapy and he has carried cases throughout their course without administering any drug. The ice bag over the heart will take the place of digitalis. He spoke of a recent case of a lady suffering great pain after having been at the dentist's. He gave her the warm baths after spine fomentations and hot foot baths and was spared the necessity of giving morphine.

Dr. J. C. White said that while he was an advocate of hydrotherapy and always had a fountain syringe as one of his greatest aids in practice, it would be bad to confine oneself to that method. Drugs had not yet lost their value when carefully considered and exhibited.

Dr. E. C. Cartledge said he frequently ordered hot water applications internally and externally in emergency cases and often found the patient almost recovered when he arrived after phoning directions as to the proper use of the water.

Dr. Neall said in closing that it was important to make the patients understand what was meant by continuous warm baths and that the temperature should be kept between 95 and 99. Often the bath would be discontinued in a short time when it was necessary to keep a patient there for two or even three or more days.

He told a lady of 50 who had been suffering with severe palpitation of the heart and convulsions following. He had kept her in the bath for three days with recovery.

He thanked the Society for the discussion.

### A SUCCESSFUL METHOD OF PERFORMING SHOCK-LESS OPERATIONS BASED ON A CLINICAL EXPERIENCE OF 3,000 CASES.

By George W. Crile, M.D.
Cleveland, Ohio.

It is the purpose of this paper to offer evidence upon which a new principle of operative surgery is based. My argument assumes that man is a motor being in the sense that Sherrington uses this phase; it assumes that physical action and emotional activity are only expressions of motor stimulation; it assumes that there are in every active animal and in man stores of energy which when released are expressed in motion or emotion; and that these stores of energy are consumed fatigue or exhaustion is produced. We shall present evidence to show that inhalation anesthesia does not wholly prevent the discharge of energy caused by an operation; that the discharge of energy of the brain cells is caused by emotional excitation, such as fear, just as readily as by physical injury—and also that the brain cells show physical changes corresponding to the exhaustion.

Turning first to the effect of physical injury, I have found that animals subjected to sufficient trauma under ether or under nitrous oxide anesthesia present a state of low vitality or surgical shock, and that their brain cells show corresponding physical changes. Under nitrous oxide, as compared with ether the animals required approximately three times as much trauma to produce an equal amount of depression of vitality, and equal physical changes in the brain cells. What is the cause of these changes in the brain cells and the loss of vitality?

First of all, let us inquire as to whether or not inhalation anesthetics act on all parts of the brain alike.

If inhalation anesthetics acted on all parts of the brain alike then the function of all the brain cells would be suspended —that is to say the patient would be dead. Beginning then with the premise that only a part of the bain is asleep under inhalation anesthesia, we may ask what is the effect on the awake brain cells? These cells respond to the physical injury of surgical operation by an effort to escape from the injury. The awake brain cells are trying to escape from the operating table and to escape from the surgeon, and these cells are just as wide awake, and are just as active as the cells of the brain in the normal wide awake state. This statement is based on the following observations:

During a surgical operation there is, in response to every incision, every pull of the retractors, indeed to every physical contact, a change in the pulse, the respiration, and the blood pressure. These changes are a part of a physical act, an effort at escape from injury. This is further shown by the fact that if the anesthesia is light and the operation is rough then the patient moves purposelessly on the operation table. It is this that leads to the exhaustion of the operation and the physical changes in the brain cells. The entire so-called subjective mind is unanesthetized, hence is fearfully punished in the course of operation.

Let us take still another viewpoint. Suppose that instead of the usual anesthesia we gave our patient curare—giving at the same time artificial respiration by intratracheal insufflation. Such a patient would not be able to move a muscle nor could he utter a sound. There would be absolute muscular relaxation, and death-like quiet during the operation, but in spite of the fact that the muscular system would be wholly paralyzed, the mind would be perfectly clear and the sense of pain normally keen.

Now what would be the effect upon a human being if a prolonged surgical operation were performed under curare? What would be the state of the nervous system of such a patient when finally he emerged from the muscle-paralyzing influence of the curare and could express his horrible experience in words? This is precisely the predicament of the subjective mind of our daily operation. It is as completely unprotected under ether as under curare—and suffers just the same.

It has been shown that animals under curare and morphia when subjected to trauma succumb to shock-producing trauma as readily as they do under ether. With these facts we can understand by what influence a strong, robust patient who enters the operating room in the full tide of health an hour later emerges broken and beaten and shattered, requiring months, perhaps years to fully recover. It is for the same reason that when run down by a railway train and mangled, a man is shattered and broken; as he is if he has passed through a horrifying experience such as that of having a pistol pressed against his forehead in the night by a highwayman; or of being the witness of a murder; or of undergoing any of the nerve-shattering stimuli of life. These and all of these are motor stimuli, and whether they impair or whether they break the nervous system their effect is just the same as the effect of surgical operations.

There is an interesting fact concerning the psychic state of the patient at the time of the operation. If the patient is in grave doubt as to whether or not he can survive the operation; if he lacks confidence in the hospital or in the surgeon, the patient has what in psychology is known as a low threshold, and if he goes under the anesthetic in this state, the effect of any physical injury will be augmented and throughout the entire anesthesia there is manifested the evidence of fear in the respiration and the pulse, and in the way in which he reacts to the anesthetic and the trauma of operation. These patients take the operation poorly. It is as though the patient went under the operation with his motor set at high speed, so that the energy of the body is consumed more rapidly, and hence the exhaustion or shock is increased.

On the other hand, we know that the field of operation is temporarily disconnected from the brain by the use of a local anesthetic, then no matter how severe, nor how extensive, nor yet how prolonged the physical injury in the zone thus blocked, no exhaustion follows, and no brain cell changes are seen.

What are the mechanisms that receive the injury impulses which are transmitted to the brain and cause a discharge of nervous energy, leading to exhaustion, to altered personality, to nervousness and all the chain of evil consequences? These mechanisms are the endings, the nerve fibres, and all of the nervous system. The nervous system acts as a unit, as a whole, and responds to but one stimulus at a time. Hence it is that if one part of the body is injured the entire nervous muscular system acts in a self-defensive manner.

Now, in the body there have been implanted innumerable nerve receptors for the purpose of effecting adaption to environment. Some of these receptors, such as those assisting in acquiring food, may be designated beneceptors, while other receptors have as their function the protection of the body against harmful or nocuous contacts. These are nociceptors. The nociceptors are not distributed over every part of the body usually, but are more numerous in those parts which were most frequently, in the course of evolution, subjected to injury.

Hence, we find in the skin the nociceptors are most numerous in those parts most exposed to contact with the outer world, viz.: in the hands and the feet; the back being less exposed has fewer nociceptors. In the deep protected parts of the body there are few nociceptors. In the brain which through all time was protected by a skull, no nociceptors are found. The brain has no pain sense. One may probe the brain of an awake patient at will without even his knowledge. It follows, therefore, that the effect of an operation in this or that portion of the body is dependent upon the phylogenetic exposure, or the number of nociceptors the part contains.

Physical injury or any sensitive area, that is, an area having nociceptors, causes a discharge of nervous energy leading ultimately to exhaustion. The exhaustion is due to forced driving of the motor mechanism. Equally well may exhaustion be produced by certain perceptions through the special senses, such as hearing or seeing a dangerous enemy.

Now, the human machine may be driven by a physical contact stimulation of the nociceptors implanted within the body, or it may be driven by perceptions through the special senses. Whatever the cause may be, the stimulus is always through the awakening of associative memory, that is, all action is on the law of association through memory, that is, through phylogenetic association.

Harmful or nocuous associations are called noci-associations. If then an operation be so planned that all harmful or nocuous associations are prevented, this state is designated anoci-association, that is to say, without noci-association. Practically applied, it means that a surgical operation performed on this principle must be so conducted that there is excluded from the brain all noci-association.

This may be accomplished as follows: The surgeon must have so thoroughly prepared himself for his work, and so controlled his surgical surroundings, that he can truthfully say to his patient that his operation will be distinctly safer than his disease; that the operation will be so conducted as to be devoid of either painful or dramatic incident, and that he will have no unpleasant experience to reflect upon afterward. The patient should be given much personal consideration by the surgeon himself; and if no contra-indication exists the patient should be given the benefits of a solacing dose of morphine, or morphia and scopolamine. The management of a patient up to the point of anesthesia should be by nurses, orderlies and physicians who are humanitarian psychologists. The anesthetist should be even more of a psychologist, and preferably a woman, as she is a more delicate recording machine. Anoci-association may be further promoted by the use of nitrous oxide which is pleasant in comparison with suffocating ether, provided only that the anesthetist is an expert in the administration of this particular anesthetic. The safety of nitrous oxide in the hands of the expert is demonstrated by the fact that in Ohio four trained anesthetists—three in Cleveland, and one in Toledo—have used nitrous oxide for general anethesia in 19,000 cases without a single anesthetic fatality.

When under anesthesia the brain may be entirely isolated from the field of operation by a careful infiltration of a solution of 1-400 novocain just as completely as if no general anesthetic had been given, and during the operation a special consideration of accuracy and gentleness should be observed. In this manner an operation, however extensive, may be performed without materially driving the motor mechanism, hence without consuming nervous energy; hence it becomes a shockless operation. At the close the zone of operation is cut off for two days from communication with the brain by an injection of quinine and urea hydrochloride, so that the after pains and post operative nerve-exhausting stimulations may be avoided.

Abdominal Operations.

1. Excluding infants, the aged, and patients with depressed vitality, we first administer, on an average, one-sixth of a grain of morphine and 1-150 of a grain of scopolamine one hour before operation.

2. If local anesthesia alone is employed, novo-caine in 1-400 solution is used by progressive local infiltration.

3. If inhalation anesthesia is employed, we then administer nitrous oxide, either alone or with ether added as required.

4. As soon as the patient is unconscious, infiltration first of the skin and then of the subcutaneous tissue with 1-400 novocain is made. In order to spread the novocain immediate local pressure with the hand is applied. Anesthesia is immediate. Incision through this anesthetized zone exposes the fascia. The fascia is then novocainized, subjected to pressure and divided. This brings us to the remaining muscle or posterior sheath and to the peritoneum, These structures are then infiltrated with novocain, subjected to pressure and divided within the blocked zone. If blocking has been complete, then upon opening the abdomen there will be found no increased intra-abdominal pressure, no tendency to expulsion of the intestines, and no muscular rigidity.

5. The peritoneum is next everted and infiltrated with ½ per cent. solution of quinine and urea hydochloride completely surrounding the line of proposed sutures and is subjected to momentary pressure. This infiltration serves as a block, and as its effects last for several days, it should prevent, or at least minimize, the post operative wound pain and the post operative gas pains, and by so much minimize post operative shock. Quinine and urea cause a certain amount of edema of tissue lasting some time after the wound is healed.

6. The relaxed abdominal wall will permit exploration of the entire abdominal cavity with ease. If in the field of operation there is no cancer and no acute infection, then the following regions may be blocked as completely and in the same manner as the abdominal wall, viz.: the meso-appendix, the base of the gall bladder, the uterus, the broad and round ligaments, the mesentery, and any portion of the parietal peritoneum. Operations on the stomach and intestines made without pulling on their attachment cause no pain, and hence require no novocain block. The closure of the upper abdomen is thus made as easy as the closure of the lower—all is done with the case of relaxation. What is the result? No matter how extensive the operation, no matter how weak the patient, no matter what part is involved, if anoci technique is perfectly carried out the pulse rate at the end of the operation is the same as at the beginning—the post operative rise of temperature, the acceleration of the pulse, the pain, the nausea, and the distension are minimized or wholly prevented.

GRAVES DISEASE.

I believe everyone will agree that a technique that can carry an advanced exophthalmic goitre case through an operation without increasing the pulse rate all the more readily do as much for any other operation. This can be done by the following technique, the operation being either ligation or lobectomy:

If ligation is made, it is performed without removing the patient from his bed. In performing ligation nitrous oxide may or may not be administered; but during the operation a complete local blocking with novocain is employed, and the entire raw field is blocked by quinine and urea hydrochloride infiltration at the close of the operation.

f lobectomy is to be performed, consent to operation is secured several days in advance, the patient being kept in ignorance of the day on which it will take place. The patient is anesthetized free from psychic strain, as he is under the impression that he is receiving an inhalation treatment.

When under anesthesia the patient is taken to the operating room. The division of tissue is preceded by a complete blocking so that no activating impulse can reach the brain. Before closing the wound a ½ per cent. solution of quinine and urea hydrochloride is infiltrated into every part of the raw field with a hypodermic needle. The patient is kept unconscious, under anesthesia, until he has returned to his room and until his room is restored to its previous condition. In the course of the cycle from his room to operation and return the brain has received no activating stimuli, and there can be no change in the pulse.

The immediate control of harmful stimuli, however, is not the end of the benefits of this operative method. The post operative hyperthyroidism is prevented or minimized, and to the same extent that the immediate results are improved so are the later clinical results improved.

What happens to a case of Graves' disease which is not under surgical treatment when subjected to a severe psychic shock—to a heavy strain or to deep worry? The disease is aggravated for weeks or for months, and not infrequently death results. The stress of facing the operating room is not only immediately seen, but is perpetuated on the following days and weeks and months by its frequent recall. From this handicap the anoci patient is free, and by so much is the convalescense speeded on its way.

### Clinical Results.

The test of any reseach, any theory, is the clinic—and the clinic only. Any theory is worthless unless it gives practical results. The clinical results I can report have been confirmed by the personal experience of a number of good clinicians—Bloodgood, Cabot and others. The work of the nurse is greatly minimized and the clinical aspect in or out of the operating room is altered. My associate, Dr. W. E. Lower, and I during the past year performed 729 abdominal sections under this method with a morality rate of 1.7 per cent; and in the Lakeside Hospital service where all kinds of acute emergencies are met, and where most of my own private work is done, there were performed by my associates and myself in the past year operations on 2,672 patients with a mortality rate of 1.9 per cent, and in the last 1,000 cases 8.1 per cent; a result never before approached in the Lakeside Hospital.

SUMMARY.

The brain being a tissue of surpassing delicacy is damaged with wonderful facility by injury and by fear and worry. The good risk when operated by almost any method by almost any surgeon of experience, will recover from his operation, but the delicate nervous organization is only too frequently shattered by the experience. We now understand why. Though the principle is clear, the technique demands to a certain extent a re-education of the surgeon; it demands a certain amount of detail and precision; it demands far more consideration for the patient; but through anoci the destiny of a patient is to a greater degree placed under the control of the surgeon, who through it is enabled to reduce both the morbidity and the mortality.

—Southern Medical Journal.

